# Obesity Increases Time to Union in Surgically Treated Pediatric Fracture Patients

**DOI:** 10.5435/JAAOSGlobal-D-21-00185

**Published:** 2022-01-05

**Authors:** David Heath, David Momtaz, Abdullah Ghali, Luis Salazar, Steven Gibbons, Grant Hogue

**Affiliations:** From the UT Health San Antonio, Department of Orthopaedics, San Antonio, TX (Dr. Heath, Mr. Momtaz, Mr. Ghali, Mr. Salazar, and Dr. Gibbons), and the Boston Children's Hospital/Harvard Medical School, Department of Orthopaedics, Boston, MA (Dr. Hogue).

## Abstract

**Introduction:**

To determine whether obesity affects time to radiographic union in surgically treated pediatric extremity fractures.

**Methods:**

A retrospective review of pediatric patients with extremity fractures at a Level 1 trauma center from 2010 to 2020. Those treated conservatively and patients with nonunions were excluded. Union was defined as radiographic evidence of bridging callus on all sides of the fracture and absence of the previous fracture line.

**Results:**

Obese patients had a markedly increased time to union when compared with others, even when age, sex, fracture type, race, and ethnicity were controlled for. The mean time to union for obese and nonobese patients were 152 and 93.59 days, respectively (*P* < 0.001). Obese patients had 3.39 times increased odds of having increased time to union. Obese patients had 6.64 times increased odds of having fractures with delayed union of 4 months or greater (*P* < 0.001).

**Conclusions:**

There is a positive correlation between obesity and time to union in surgically treated pediatric fracture patients.

Childhood obesity is a public health issue that has reached alarming rates in the 21st century.^1^ Childhood obesity has been studied extensively as an important risk factor for various medical pathologies such as type 2 diabetes and coronary artery disease.^1,2^ The Center for Disease Control and Prevention (CDC) defines obesity as body mass index (BMI) at the 95th percentile and above based on age and sex-matched parameters. The prevalence of obesity for children and adolescents aged 2 to 19 in 2017 to 2018 was 19.3%. Among different populations, childhood obesity is most prevalent in Hispanic and non-Hispanic Black populations at 25.6% and 24.2%, respectively.^3^ Lee et al^4^ presented a novel study that investigated the impact of obesity on fracture healing, showing obese/overweight children did not have a delay in release to activity when compared with normal weight children. The authors identified a limitation of their study in that this outcome is based on subjective interpretation of the patient's clinical picture. Limited efforts have demonstrated objective findings to study fracture healing and obesity.

Obesity increases the incidence of fractures and poor mobility in children.^5-7^ Obesity may also be a risk factor for sustaining an extremity fracture requiring surgical intervention with a higher risk for certain postoperative complications. Biochemically, osteoblasts and adipocytes are derived from a common mesenchymal stem cell. In obesity, these cells may show preference to differentiate to adipocytes rather than osteoblasts, decreasing bone formation.^8^ In addition, obesity increases the amount of proinflammatory markers such as osteoprotegerin ligand, which promotes osteoclast differentiation and activation, increasing bone resorption.^9^ In the digestive tract, increased fat intake may interfere with calcium absorption in the gut because of binding with fatty acids, thus decreasing the availability of calcium for bone formation.^10^

Although previous investigations have examined obesity as a risk factor for fracture and in return to activity, there is a paucity of literature examining objective findings for fracture healing based on radiographic evidence. The purpose of this study was to investigate radiographic time to union between different cohorts based on BMI classification. The CDC guidelines for the definition of each classification were used. Less than the fifth percentile on the BMI-for-age growth chart is considered underweight. Fifth to 85th percentile is considered normal weight. 85th to 95th percentile is overweight. 95th percentile or greater is obese.^11^ The authors hypothesize that patients in the obesity cohort will exhibit longer periods to reach radiographic union.

## Methods

This retrospective review was approved by the Institutional Review Board at our Level 1 accredited pediatric trauma center. We collected data via retrospective chart review for patients between ages 10 and 18 years of age and treated for common fractures between January 2010 and June 2020. Nonextremity fractures and those treated conservatively were excluded from our study. Conservative fractures were excluded because of their propensity to have less follow-up, with most not having radiographic evidence of union before being lost to follow-up. In addition, those who underwent revision surgery for reasons other than hardware removal and those who did not follow-up to radiographic union were excluded from the study. Demographic and medical information were collected that included variables such as BMI, age, race, date of injury, location of injury, surgery indicated, surgical complications, and radiographic time to union. Based on Heath et al,^12^ radiographic union was defined as bridging bone at the fracture site on all cortices with resolution of the fracture line. Initial chart review was performed by three medical students; however, both the staff pediatric orthopaedic surgeon and orthopaedic surgery resident on the research team confirmed all radiographic image findings to ensure accuracy of calculated time to union.

The final number of patients included in the study was 147, with five underweight, 81 normal, 33 overweight, and 28 obese BMI patients as defined by the CDC. Stratification was done with consideration to the patient's age and sex. Statistical analysis compared BMI against time to union with sex, age at the time of surgery, race, and ethnicity serving as control subject variables. The sample contained no tobacco, alcohol, or other illicit drug users identified on urine drug screen. The collected variables were then analyzed using commercially available software packages. Microsoft Excel was used to sort the data and perform preliminary analysis. International Business Machines Statistical Package for the Social Sciences suite was used to run analysis of variance, independent samples T tests, logistic regressions, and Fisher exact tests, as well as a number of cross-tabulations and descriptive statistics. Data were analyzed first to ensure all variables met the criteria of the respective test being done. For analysis of variance results, data met the assumption of homogeneity of variances and were post hoc tested with the Tukey test at an alpha of 0.05. Logistic regression-dependent variables were analyzed to ensure little to no multicollinearity and independence of observations. Furthermore, linearity of independent variables and log odds was observed. All variables in multivariable logistic regression models were first ran separately to ensure no artifact *P* values were present and that all effect sizes were reported honestly and not overinflated because of interaction. No notable interactions were seen between independent variables. Confidence intervals were set at 95% with a *P* value of 0.05 being considered statistically significant.

## Results

A total of 459 pediatric patients underwent surgical treatment for fractures of extremities by surgeons at our institution. After the inclusion and exclusion criteria, 147 patients from 2010 to 2020 met all inclusion criteria and were stratified into groups based on their BMI percentiles. Our sample contained five underweight, 81 normal weight, 33 overweight, and 28 obese patients. There were 10 Black and 53 Hispanic patients. There was a predominance of men in the overall group (66.7% male versus 33.3% female). The mean age for the sample was 11.83 years, with the youngest patient just younger than 3 years old and the oldest just older than 17 years of age. These and other sample demographics can be found below in Table 1. Fracture frequencies by type along with their Orthopaedic Trauma Association classifications are listed in Table 2. No patients had any superficial or deep wound complications.

**Table 1:** Sample Demographics and Descriptive Statistics

Condition	N (%)
Underweight	5 (3.40)
Healthy	81 (55.10)
Overweight	33 (22.40)
Obese	28 (19)
Male	98 (66.70)
Female	49 (33.30)
Non-Black	137 (93.20)
Black	10 (6.80)
Non-Hispanic	94 (63.90)
Hispanic	53 (36.10)
Condition	Mean (SD)
BMI	21.44 (5.21)
Time to union (d)	104.71 (82.37)
Age (yr)	11.83 (3.28)
BMI percentiles	70.19 (27.99)

BMI = body mass index

**Table 2:** Fracture Frequencies and OTA Classifications for all Patients (N = 147)—Stratified by Obese (N = 28) vs Other (N = 119)

	Other		Obese	
Fracture	N (%)	OTA Other	N (%)	OTA Obese
Ankle	2 (1.68)	44B3.3, 44B3	2 (7.14)	44C2, 44C2.2u
Femur	33 (27.73)	32A1b, 31A, 31A1, 31A2, 32-C3, 32A1b, 32A3b, 32B3, 32B3b, 32C2, 32C3j, 33A2.2, 33B1, 32A3a, 2x (32A1, 33A2), 3x (32C3), 4x (32A2, 32A3)	7 (25.0)	33A2, 32A3a, 33A2.3, 32A2, 3x (32A3)
Fibula	6 (5.04)	4F2Ac, 5x (4F2A)	3 (10.71)	4F2A, 2x (4F2B),
Heel	1 (0.84)	82B	0 (0.00)	
Humerus	22 (18.49)	11A2.1, 11B1, 11C1, 12A1, 12A3, 13A2, 13A3.1, 13A3.2, 13C2, 2x (12C3, 13A1, 13B1, 13C1, 11A2), 3x (13C).	6 (21.43)	13C1, 2x (11A2), 3x (12C2j)
Iliac crest	1 (0.84)	61A2.1	0 (0.00)	
Metacarpal	2 (1.68)	2x (77.3.2A)	0 (0.00)	
Phalanx	6 (5.04)	78.5.1.2C, 2x (78.5.1.1A), 3x (78.2.1)	1 (3.57)	78.5.1.2C
Radius	16 (13.45)	2R1B1, 2R1C1, 2R2A1a, 2R2B3, 2R3A, 2U2C3, 42A3b, 2R3B1, 2x (2R3A2.1, 2R2A3), 4x (2R3A2)	6 (21.43)	2R3, 2R3C2.1, 2R3B1, 2R2A1, 2x (2R2A3)
Talus	2 (1.68)	81.1.B3, 81.2.B	0 (0.00)	
Tibia	17 (14.29)	41A2.2, 42A1, 42A2b, 42C3, 43A1, 43A3.3, 42C2, 2x (42A1, 42A3, 43C1, 4F2B), 3x (42A2)	1 (3.57)	42C2
Ulna	11 (9.24)	2U2A2a, 2U3A2.3, 2U3A, 2U3B, 2U2A2, 2U3, 2U2A2b, 2U2B3, 2R3A3.3, 2x (2U3A2)	2 (7.14)	2U3, 2U2A2

OTA = Orthopaedic Trauma Association

Statistical models were created and run with obesity serving as the independent variable and time to union as the dependent variable. Sex, age, fracture type, race, and ethnicity were used as controls. No statistical significance existed in the relationship between sex and time to union. Likewise, there was no association between age at the time of surgery, race, or ethnicity when it came to predicting time to union. The impact of obesity on time to union remained notable despite controlling for all the aforementioned variables.

**Figure 1:**
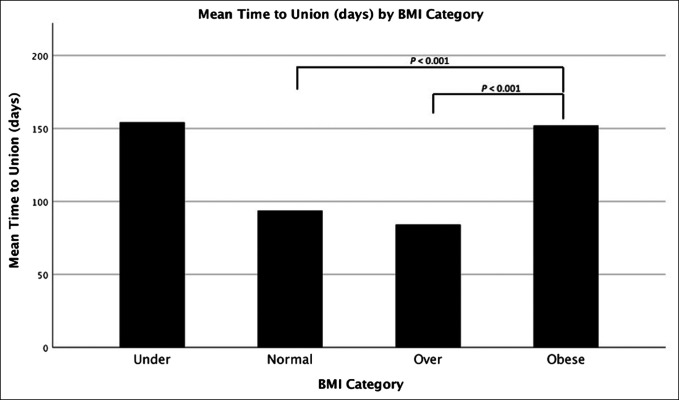
Chart showing the mean time to union in days for respective body mass index categories. Mean time to union for obese patients was significantly longer when compared with normal weight and overweight patients (*P* < 0.001). Underweight patients demonstrated a larger mean time to union, but this result was not statistically significant likely due to being underpowered (n = 5).

A further model was analyzed to elucidate the significance of mean time to union between the CDC BMI categories in our sample. The mean time to union for each category (154.2 days for underweight, 93.69 days for normal weight, 84.15 days for overweight, and 152 days for obese) was compared against the others to determine statistical significance or lack thereof. The mean time to union for obese patients was statistically significant when compared with normal weight and overweight patients (*P* < 0.001). Underweight patients demonstrated a larger mean time to union; however, this result was not statistically significant likely because of being underpowered. All other comparisons between each BMI category were not statistically significant. These results are displayed in Figure 1.

**Figure 2:**
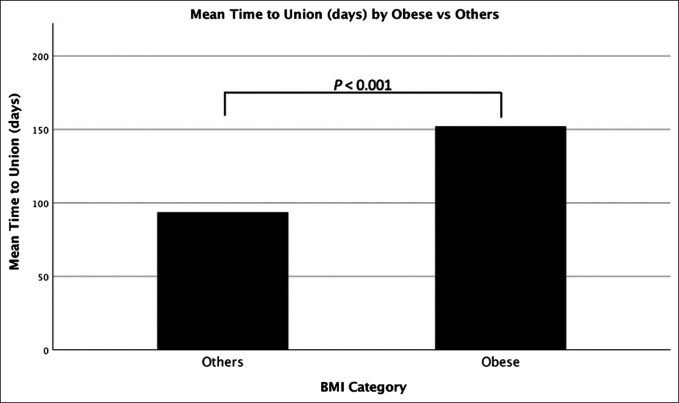
Chart showing the mean time to union in days comparing obese patients and all others. Obese patients had a mean time to union of 152 days, whereas those in all other categories had a mean time to union of 93.59 days (*P* < 0.001).

The mean time to union was assessed between obese patients and all others. It was found that obese patients had a mean time to union of 152 days, whereas those in all other categories had a mean time to union of 93.59 days (*P* < 0.001). These results are displayed in Figure 2.

Further analysis revealed obese patients had an odds ratio of 3.49 to have an increased time to union when compared with others (*P* = 0.005). An increased time to union was defined as a time to union that exceeded the sample median of 79 days. The median was selected for its inherent ability to account for outliers. These results are further displayed in Table 3.

**Table 3:** Proportion Comparison Obese and Increased Time to Union

Condition	Normal Time to Union	Increased Time to Union
Others	64	55
Obese	7	21
Total	71	78
*P* = 0.005		

Furthermore, we cross-tabulated normal weight patients for having a normal time to union and found that they had an odds ratio of 6.83 to have a normal time to union when compared with others (*P* = 0.007). When we controlled for age, sex, fracture type, race, and ethnicity, we continued to observe an increased odds of 2.48 for normal weight patients to have a normal time to union (*P* = 0.008).

Finally, we controlled for age, sex, fracture type, race, and ethnicity and ran a series of calculations. We found that obese patients, on average, had an increased time to union by 68.37 days when compared with others (*P* < 0.001). Obese patients had 3.39 times increased odds of having an increased time to union (*P* = 0.01). Obese patients had 6.64 times increased odds of having a time to union, which exceeded 4 months (*P* < 0.001).

## Discussion

According to the World Health Organization, childhood obesity is a global problem, with its prevalence increasing four-fold over the last 25 years.^11,13^ Beyond its deleterious effects in the cardiopulmonary system, obesity has been associated with notable orthopaedic risks as well. Preoperatively, obesity has been shown to increase the risk of fractures and fracture severity, and the complexity of the fracture type.^14-16^ Intraoperatively, a larger body habitus can make surgical procedures more technically challenging because anatomical landmarks are harder to delineate and can cause longer surgical times for patients.^7^ Postoperatively, childhood obesity has been linked to increased risk of nerve palsies, wound infection, wound dehiscence, delayed healing, and refracture through an old fracture site.^15,17^ Our study is the first to evaluate the effect of childhood obesity on time to radiographic union, defined as bridging callus on all sides of the fracture and resolution of the previous fracture line, in surgically treated pediatric fracture patients. This study demonstrates a markedly increased time to union for overweight and obese children compared with normal-weight children for surgically treated patients. Overweight or obese children were 2.65 times more likely to have an increased time to union than normal-weight patients. Notably, our study also found that obese patients took 58 more days on average to unite their fractures than normal-weight children.

Previous studies have shown notable associations between obesity and fracture-healing risk. Zura et al^18^ presented an inception cohort study analyzing fracture nonunions in more than 300,000 fractures with a 1-year follow-up. They found a 1.2-fold increased risk of nonunion in adults with obesity than a person without obesity. Similarly, in a systematic review evaluating the biological risk factors for nonunion, Zura et al.^19^ found that obesity increased the risk of nonunion in six of the eight studies they included. These results were isolated to studies performed in the humerus, femur, and tibia, with most patient populations represented being adults. A more recent meta-analysis of tibial nonunions confirmed that obesity and diabetes were the most notable negative influences on fracture healing.^20^ In contrast, our study evaluates fracture time to union in all surgically treated pediatric extremity fractures. Furthermore, Tian et al. defined tibial nonunion as no sign of union within 9 months of operation, whereas our definition focuses on radiographic findings. In the largest study evaluating fracture nonunion risk in the pediatric population, Zura et al^21^ established BMI as a risk factor for nonunion in their cohort study of more than 200,000 fractures with a 1-year follow-up. However, their study's identification of nonunions was based on patients having the International Classification of Diseases, Ninth Revision, code for nonunion correctly documented in their chart. This could introduce imprecision, coding errors, and variability between surgeons' documentation of nonunion as a precise and reproducible definition of nonunion is difficult.^22,23^ Our study takes a different approach, defining union on the basis of radiographic markers that could be easily replicated and validated by future studies.

Although this study focuses on the effects of pediatric obesity on time to union, obesity also has notable negative associations to fracture-healing in general that are becoming increasingly recognized in the orthopaedic literature. This may be related to metabolic changes associated with obesity that inhibit osteogenic and hematopoietic regenerative factors.^24,25^ However, the exact relationship between obesity and bone metabolism remains nebulous and necessitates further studies to fully elucidate.^23^ Nevertheless, studies have shown that obesity also increases the risk of postoperative complications. Moroz et al^26^ showed children over 49 kg were five times more likely to have negative sequelae after titanium elastic nailing of their femur fracture than those weighing less than 49 kg. Mehlman and Bishai^27^ demonstrated that children over 45 kg predicted malunion after surgery for their femoral shaft fracture. Weiss et al.^28^ reported that obese children were two times more likely to develop a wound or hardware complication when undergoing flexible elastic nailing for femur fractures. This study compares favorably with these findings. In our study population, obese patients had 3.49 times increased odds of having an increased time to union compared with the sample mean. Obese patients had 6.64 times increased odds of having delayed union of their fracture to 4 months or greater. Our results add to the growing body of evidence suggesting the negative impact of obesity in the pediatric orthopaedic patient. These findings may serve as a reference for orthopaedic surgeons while attempting to promote healthy lifestyle choices with their patients and helps in preoperative counseling when discussing return to activities or sport. This also helps orthopaedic surgeons in preoperative counseling and when discussing return to activity.

The main strength of our study is that it uses a clear and objective definition of union, whereas current literature on the determination of union varies markedly and introduces variability subjective to surgeon preference. Our study contributes a concrete and reproducible analysis of the effect of obesity on union and healing. However, our study is not without limitations. Retrospective studies have biases inherent to the data retrieval and interpretation processes that may alter our findings. In addition, our patient population data were collected from a single institution, limiting external validity. Another limitation of our study is the omission of nonunions. In our study design, we excluded patients who were reoperated on for reasons other than hardware removal, likely removing many nonunions before radiographic review. We then identified no nonunions among those patients whose radiographs were reviewed. Owing to the design of our study likely underestimating the number of nonunions, we chose to omit them altogether. This is an area for further exploration in future studies. Our study is unique in that it focuses on those patients who had fractures that went on to union, and we implemented a strict, objective definition of union, rather than relying on International Classification of Disease coding or mention of union in progress notes. Furthermore, a potential weakness in our study is not accounting for soft tissue envelope-specific injuries to differentiate between open or closed fractures. Finally, our study also did not control subject for varying activity levels such as preinjury activity and medical comorbidities such as type 2 diabetes, which may confound healing patterns in the pediatric population. Future studies that control for these factors are indicated.

## Conclusion

In conclusion, there is a notable correlation between obesity and time to union in surgically treated fractures in pediatric patients. This is the first study to evaluate the effects of obesity on radiographic time to union in surgically treated pediatric fracture patients using a strict, objective definition of union based on direct radiographic review. Obese patients have 3.39 times increased odds of delayed time to unite fractures compared with their normal weight counterparts even when age, sex, fracture type, race, and ethnicity were controlled for. Further prospective trials comparing overweight or obese children with normal-weight children are necessary to confirm these findings (http://links.lww.com/JG9/A179).
